# Effects of Anti-CGRP Monoclonal Antibodies on Neurophysiological and Clinical Outcomes: A Combined Transcranial Magnetic Stimulation and Algometer Study

**DOI:** 10.3390/neurolint16040051

**Published:** 2024-06-22

**Authors:** Paolo Manganotti, Manuela Deodato, Laura D’Acunto, Francesco Biaduzzini, Gabriele Garascia, Antonio Granato

**Affiliations:** 1 Department of Medical, Surgical and Health Sciences, University of Trieste, 34100 Trieste, Italy; pmanganotti@units.it (P.M.); laura.d.acun@gmail.com (L.D.); alterxx92@hotmail.it (F.B.); gabrielegarascia@gmail.com (G.G.); antonio_granato@hotmail.com (A.G.); 2 Azienda Sanitaria Universitaria Giuliano Isontina (ASU GI), 34128 Trieste, Italy

**Keywords:** migraine, cortical excitability, anti-CGRP monoclonal antibodies, transcranial magnetic stimulation, motor cortex, trigeminocervical complex

## Abstract

Background: the aim of this study was to investigate the neurophysiological effect of anti-CGRP monoclonal antibodies on central and peripheral levels in migraine patients. Methods: An observational cohort study in patients with migraine was performed. All subjects underwent Single-Pulse and Paired-Pulse Transcranial Magnetic Stimulation, as well as a Pressure Pain Threshold assessment. The same protocol was repeated three and four months after the first injection of anti-CGRP monoclonal antibodies. Results: A total of 11 patients with a diagnosis of migraine and 11 healthy controls were enrolled. The main findings of this study are the significant effects of anti-CGRP mAb treatment on the TMS parameters of intracortical inhibition and the rise in the resting motor threshold in our group of patients affected by resistant migraine. The clinical effect of therapy on migraine is associated with the increase in short-interval intracortical inhibition (SICI), resting motor threshold (RMT), and Pressure Pain Threshold (PPT). In all patients, all clinical headache parameters improved significantly 3 months after the first injection of mAbs and the improvement was maintained at the 1-month follow-up. At baseline, migraineurs and HCs had significant differences in all TMS parameters and in PPT, while at follow-up assessment, no differences were observed on RMT, SICI, and PPT between the two groups. After anti-CGRP monoclonal antibody injection, a significant increase in the intracortical inhibition, in the motor threshold, and in the Pressure Pain Threshold in critical head areas was observed in patients with migraine, which was related to significant clinical benefits. Conclusions: Anti-CGRP monoclonal antibodies improved clinical and neurophysiological outcomes, reflecting a normalization of cortical excitability and peripheral and central sensitization. By directly acting on the thalamus or hypothalamus and indirectly on the trigeminocervical complex, treatment with anti-CGRP monoclonal antibodies may modulate central sensorimotor excitability and peripheral sensitization pain.

## 1. Introduction

Migraine represents one of the most severe and prevalent brain conditions [1]. Its physiopathology is characterized by two opposing processes, habituation and sensitization, that together determine the cycle of a migraine attack [2]. Indeed, migraine attacks present with two main phases, namely the prodromal and the headache phases. The prodromal phase is associated with a lack of habituation, which may be neuro-physiologically characterized by alterations both in cortical excitability and in intracortical circuits. The headache phase is associated with sensitization, possibly related to a reduction in Pressure Pain Threshold (PPT). Consequently, habituation and sensitization are considered fundamental in studying central and peripheral systems involved in migraine and in understanding the network modified by the treatments [2,3,4].

Extensive neurophysiological studies in migraineurs have recognized neurological lesions and damage [5,6], biomarkers associated with inflammation [7,8], and abnormal information processing and functional connectivity [9], involving habituation and sensitization opposing processes [4] that change during the migraine phases [10,11]. Habituation consists of a reduction in response to sensory stimulation, while sensitization consists of an increase in response to sensory stimulation. On the one hand, a lack of habituation leads to an abnormal state of cortical excitability characterized by an alteration, whose value stands between the threshold of motor/occipital cortex activation and that of inhibitory/excitatory intracortical circuits. On the other hand, sensitization leads to an abnormal state of responsiveness of central and peripheral neurons among the trigeminocervical complex and brain areas [2,3,4].

Transcranial Magnetic Stimulation (TMS) has been largely investigated in migraine patients [12,13]. Increases in cortical excitability and alterations in the inhibitory/excitatory intracortical circuits were largely reported in migraineurs [14,15]. On the other hand, sensitization at the peripheral level could be assessed with PPT. In particular, pressure algometry allows the evaluation of the sensitivity of tissues both over the muscles in the trigeminocervical area and over the muscles in the extra-trigeminocervical area [16,17,18].

The trigeminocervical complex seems to be at the center of the interplay between the central and peripheral structures involved in migraine, such as the intra- and extracranial blood vessels, upper cervical spinal cord, locus coeruleus, periaqueductal grey, hypothalamus, primary and secondary motor cortex, somatosensory and visual cortex, thalamus, insula, and amygdala [3,4]. The activation of the trigeminocervical complex connects peripheral events with central involvement through the release of different neuropeptides. Among these, the Calcitonin Gene-Related Peptide (CGRP) is considered the principal mediator of migraine. It is widely distributed in the central and peripheral nervous systems. Blood levels of CGRP are higher in migraine patients compared to healthy controls (HCs) both during the pain and the interictal phases; further, they are higher in Chronic Migraine (CM) compared to Episodic Migraine (EM) patients [19,20,21]. For this reason, the emerging treatment options against CGRP signaling have shown encouraging results in relieving migraine attacks.

Four monoclonal antibodies (mAbs) targeting the CGRP pathway have been approved for the prevention of migraine attacks in EM and CM in adults: erenumab, fremanezumab, galcanezumab, and eptinezumab. Fremanezumab, galcanezumab, and eptinezumab directly target CGRP, whereas erenumab acts as a blocker of CGRP receptors. Several studies have investigated the clinical effectiveness of anti-CGRP mAbs on headache frequency, duration, and intensity [22,23]. A comprehensive review of phase II–III RCTs involving the four anti-CGRP mAbs has shown that these molecules are significantly superior to placebo in both EM and CM [24]. Ongoing real-world studies are confirming these data, including refractory patients [25]. One study found a mild influence of erenumab 70 mg exclusively on trigeminal districts [26]. However, no previous studies have investigated whether the direct site of action of mAbs is exclusively peripheral or may also include central targets.

The main aim of this study was to investigate the effect of anti-CGRP mAbs on central and peripheral levels in migraine by the neurophysiological evaluation of cortical excitability and PPT. The secondary aim was to compare the neurophysiological outcomes with those of healthy subjects after mAb treatment.

## 2. Materials and Methods

This observational cohort study was performed in patients with migraine without aura according to the International Classification of Headache Disorders (ICHD-3) [27] criteria. This study was conducted in accordance with the Declaration of Helsinki and the project was approved by the relevant institutional review board and ethics committee (CEUR-2021-Os-246; ID 4174). Moreover, all patients signed the informed consent. Patients with migraine were enrolled at the tertiary Headache Centre of the Clinical Unit of Neurology from March 2021 to June 2021.

Patients were treated with anti-CGRP mAbs according to the AIFA (Italian Medicines Agency) criteria for anti-CGRP monoclonal antibody prescription (erenumab, galcanezumab, and fremanezumab; eptinezumab was not available in Italy). These criteria included the following: migraine diagnosis with more than 8 days per month of migraine in the last 3 months; MIDAS (Migraine Disability Assessment Score) > 11; and failure or contraindication of at least three classes of preventive drugs, including β-blockers, antiepileptics, antidepressants, and Onabotulinumtoxin-A. The exclusion criteria were as follows: patients <18 and >65 years old; pregnancy and breastfeeding; contraindications or low tolerance to TMS; other neurological or psychiatric disorders; cranial nerve impairment; cardiac implantable devices; current prophylactic treatments with antiepileptic drugs and/or benzodiazepines; other migraine prophylactic treatments in the past 3 months; and patients who did not provide their consent to this study. Failure of previous prophylaxes was defined as a treatment discontinuation due to unacceptable side-effects and/or to the absence of improvement in headache after a period of 6 weeks of therapy.

The type and doses of CGRP mAbs were determined according to the clinical evaluation of a headache expert and tailored on migraine patients’ characteristics. The therapeutic doses subcutaneously administered were as follows: erenumab 140 mg/28 days dose; fremanezumab 225 mg/month dose; and galcanezumab 120 mg/month dose with a single loading dose of 240 mg. The control group included HCs recruited among resident doctors and health care practitioners between 18 and 65 years old who met the same exclusion criteria while not suffering from headache.

The migraine group was defined using the following frequency features: High-Frequency Episodic Migraine (HFEM) = 8–14 headache days per month, calculated as ≥8 to ≤14; CM ≥ 15 headache days per month for more than 3 months with at least 8 days with features of migraine headache (ICHD-3 criteria) [27].

### 2.1. Study Design

At baseline (t0), before starting anti-CGRP monoclonal antibody therapy, all patients underwent Single-Pulse (SP) and Paired-Pulse (PP)-TMS and PPT assessment. The same protocol was repeated three (t1) and four (t2) months after the first injection of anti-CGRP monoclonal antibodies. The control group underwent the same protocol with SP-TMS, PP-TMS, and PPT assessment only once (t0). All evaluations were performed during the pain-free periods (i.e., at least 3 days after the last day of migraine and 2 days before the following one) [28] and, for female patients, only in the late follicular phase (i.e., between the day following the end of the menstrual cycle and the day before the start of the ovulation) [29]. Furthermore, subjects should not have taken any medications in the 72 h before the TMS and PPT assessment [30]. The frequency of migraine, duration of attacks, and drug intake of each patient were collected in the headache diary and the MIDAS questionnaire was performed at t0 and t1.

### 2.2. Neurophysiological Parameters

#### 2.2.1. Transcranial Magnetic Stimulation

SP and PP-TMS sessions were performed in line with the International Federation of Clinical Neurophysiology guidelines and well-established protocols adopted in previous studies to test the cortical excitability of migraine patients [31,32]. Subjects remained sitting in a quiet room, resting with open eyes. Stimuli were delivered by a stimulating figure-of-eight coil of a MagPro^®^ magnetic stimulator (MagVenture Inc., Alpharetta, GA, USA) connected to an electromyographic device (Synergy^®^, Natus^®^, Middleton, WI, USA). The optimal site corresponding to the left motor cortex was identified by making patients wear a tight-fitting plastic swimming cap, in order to precisely elicit responses of the contralateral abductor pollicis brevis (APB) muscle in each subject [31]. The low frequency was set at 3 Hz and the high frequency was set at 10 kHz. The electromyographic signals were recorded using Ag-AgCl surface electrodes on the right hand, with a bandpass of 10 to 1000 Hz and a display gain ranging from 50 to 1000 μV/cm. The active (cathode) electrode was placed on the APB muscle, the reference electrode (anode) on the first proximal phalanx, and the ground electrode distally on the volar surface of the forearm. The background electromyographic activity was monitored and recorded to determine the state of muscle relaxation.

The following parameters were obtained for each patient:

1. The resting motor threshold (RMT), from SP-TMS, is defined as the minimum stimulation intensity required to produce a peak-to-peak motor-evoked potential (MEP) amplitude of ≥50 μV in at least five of ten stimulations.
2. A short-interval intracortical inhibition (SICI), from the PP-TMS session, evoked by delivering a subthreshold (80% RMT) Conditioning Stimulus (CS) followed by a suprathreshold (130% RMT) test stimulus (TS) at interstimulus intervals (ISIs) of 3 and 5 ms.
3. Intracortical facilitation (ICF), from the PP-TMS session, with the same CS (80% RMT) and TS (130% RMT) at longer ISIs of 10 ms, 15 ms, and 20 ms.
4. Eight MEPs were recorded from the SP-session, elicited by delivering a suprathreshold (130% RMT) TS.

Eight trials were recorded from the SP-TMS, while four trials were delivered for each ISI during the PP-TMS session. The amplitude of motor-evoked potentials (MEPs) elicited by single or paired magnetic stimuli was calculated peak-to-peak and then averaged for each stimulation intensity. From the PP-TMS session (SICI and ICF), the MEP variation was calculated as the mean percentage of the ratio between the MEP obtained by the conditioned stimulus and the basal MEP [15].

#### 2.2.2. Pressure Pain Threshold

For PPT to be evaluated, the Somedic algometer was chosen for its reliability and validity [17,18,33]. The small surface of the Somedic algometer guaranteed an accurate assessment of the craniofacial muscles. The procedure was performed in accordance with Andersen’s guidelines of the PPT assessment standardization over craniofacial muscles [33]. PPT was assessed bilaterally over five muscles of the trigeminocervical complex (i.e., masseter, temporalis, trapezius, sub-occipitalis, and procerus) and over one muscle far from this area (i.e., tensor fasciae latae). The first application was conducted on the wrist of each subject, so they could familiarize themselves with the procedure. Three consecutive measurements were carried out for each muscle, with a one-minute interval between each measurement and following the same order of application to the muscles. The increasing rate was approximately 30 kPa/s. When the feeling of pressure turned into pain, patients had to press the algometer stop button [33].

### 2.3. Headache Parameters

Each patient was given a headache diary for them to record the days of the month of migraine, the duration of pain, and the symptomatic drug intake. Patients were asked to take symptomatic medications only in case of severe headache with a limit of twice per week. At t1 and t2, patients who showed a ≥50% reduction from baseline in the days of the month of migraine were considered responders, and those who showed a ≥75% reduction were considered super-responders. Disability was evaluated with MIDAS.

### 2.4. Statistical Analysis

Data were analyzed with GraphPad InStat 3.06. The statistical significance level was ɑ 95% (0.05). The Mann–Whitney Test was used to compare data of migraine patients with data of the HCs, while the Friedman Test (Nonparametric Repeated-Measures ANOVA) was used for establishing the differences among the three patient evaluations. The graphical representation of data was performed with GraphPad Prism 8.4.1.

## 3. Results

A total of 22 subjects were enrolled, 11 patients with migraine and 11 HCs. Migraineurs comprised 3 men and 8 women, with a mean age of 45 years (SD ± 13). At the time of enrollment, 6 patients met the CM criteria, while 5 patients suffered from HFEM (Table 1). The HC group consisted of 5 men and 6 women, with a mean age of 41 years (SD ± 13). No differences were found between the two groups at t1 in terms of age (*p* = 0.4).

### 3.1. Transcranial Magnetic Stimulation

#### 3.1.1. SP-Protocol: Resting Motor Threshold

**Figure 1:**
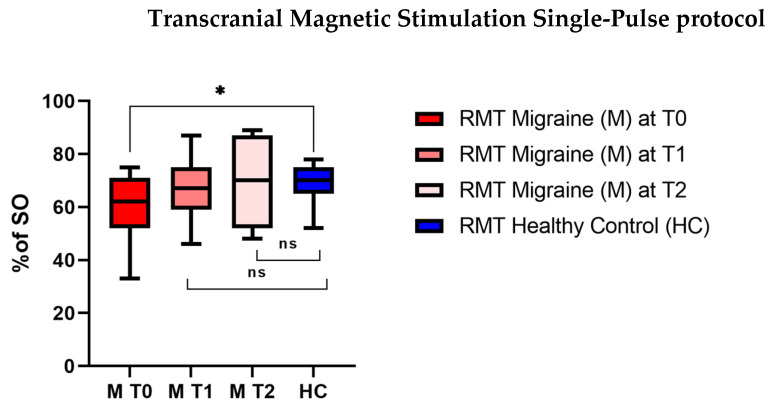
Resting motor threshold (rMT, % of stimulator output (SO)) of individuals with migraine at baseline (t0), at the end of 3 months of therapy with monoclonal antibodies (t1), and after one month of follow-up (t2) and resting motor threshold (rMT, % of stimulator output (SO)) of healthy controls. Mann–Whitney Test and Friedman Test (Nonparametric Repeated-Measures ANOVA): * *p* < 0.05.

At t0, HCs had a mean RTM (68.6; SD ± 8.1) significantly higher (U = 91; *p* = 0.04) than that of migraineurs (59.2; SD ± 12.3), but the differences at t1 (*p* = 0.5) and t2 (*p* = 0.8) between the two groups were not significant. Indeed, at t1 and t2, RMT increased in the migraine group to 66 (SD ± 12.3) at t1 and to 70 (SD ± 15.8) at t3. Moreover, the Friedman Test revealed statistically significant differences (^χ2^_F_(2) = 9.000; *p* = 0.01; Kendall’s W = 0.8610) among the three evaluations (t0 vs. t1—6.000, ns *p* > 0.05; t0 vs. t2—12.000, * *p* < 0.05; t1 vs. t2—6.000, ns *p* > 0.05) (Figure 1) (Table 1).

**Table 1:** Demographic and clinical data of migraine group.

	Gender	Age	Diagnosis	Previous Prophylactic Therapy	mAbs	MMDs			Duration			MDI		MIDAS		
						t0	t1	t2	t0	t1	t2	t0	t1	t2	t0	t1
Patients																
1	F	46	CM	Am; BoNTA; Tiz; Top	Er	20	5	6	163	24	47	20	12	7	124	28
2	F	22	CM	Am; BoNTA; Er; Flu; Prop; Top	Er	20	19	16	526	123	104	5	4	5	126	52
3	F	62	CM	Am; BoNTA; Flu; Prop; Top; Ven	Er	20	1	2	518	6	14	12	2	1	114	4
4	F	61	CM	Am; Flu; Prop; Top; VPA	Gal	15	5	4	105	30	14	25	4	3	120	19
5	M	41	HFEM	Am; BoNTA; Er; Flu; Prop; Top; VPA	Er	10	9	5	52	49	45	12	15	14	64	31
6	M	42	HFEM	Am; Flu; Top	Er	14	8	9	42	40	48	14	7	6	84	25
7	F	65	CM	Am; Preg	Er	18	11	7	92	70	29	24	11	8	114	32
8	F	47	HFEM	Am; Flu; Top	Fre	12	10	8	141	87	80	9	6	4	61	30
9	F	33	HFEM	Top	Er	12	6	3	45	14	6	12	10	3	86	6
10	M	49	CM	BoNTA; Met; Top;VPA	Fre	19	7	4	85	29	11	30	9	9	131	7
11	F	34	HFEM	Am; BoNTA; Top; VPA	Fre	12	6	5	37	15	8	12	6	7	78	2
Mean (SD)		45 (±13)				15 (±3)	7 (±4)	6 (±3)	164 (±181)	44 (±35)	36 (±31)	15 (±7)	7 (±3)	6 (±3)	100 (±25)	21 (±15)
Median (IQR)		46 (5.5–55)				15 (12–19.5)	7 (5.5–9.5)	5 (4–7.5)	92 (48.5–152)	30 (19.5–59.5)	29 (12.5–47.5)	12 (12–22)	7 (5–10.5)	6 (3.5–7.5)	114 (81–122)	25 (6.5–30.5)
Difference																
t0 vs. t1						*p* ≤ 0.01			*p* ≤ 0.05			*p* = ns		*p* ≤ 0.0001		
t0 vs. t2						*p* ≤ 0.001			*p* ≤ 0.01			*p* ≤ 0.01		-		
t1 vs. t2						*p* = ns			*p* = ns			*p* = ns		-		

Am: Amitriptyline; BoNTA: Onabotulinumtoxin-A; CM: Chronic Migraine; Er: erenumab 140 mg; Fre: fremanezumab; Flu: Flunarizine; Gal: galcanezumab; HFEM: High-Frequency Episodic Migraine; mAbs: monoclonal antibodies; Met: Metoprolol; MIDAS: Migraine Disability Assessment Score; MDI: Monthly Drug Intake; MMDs: Monthly Migraine Days; Preg: Pregabalin; Prop: Propranolol; Tiz: Tizanidine; Top: Topiramate; VPA: Valproic acid; Ven: Venlafaxine.

#### 3.1.2. PP-Protocol of TMS: Intracortical Inhibition and Intracortical Facilitation

**Figure 2:**
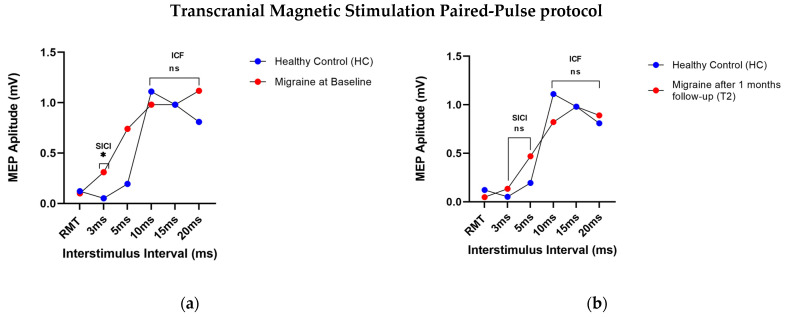
Transcranial Magnetic Stimulation (TMS) Paired-Pulse protocol: (**a**) TMS motor-evoked potentials (MEPs) at different interstimulus intervals (ISIs) of individuals with migraine at baseline (t0) and of healthy controls. Mann–Whitney Test and Friedman Test (Nonparametric Repeated-Measures ANOVA): * *p* < 0.05. (**b**) TMS motor-evoked potentials (MEPs) at different interstimulus intervals (ISIs) of individuals with migraine after one month of follow-up (t2) and of healthy controls. Mann–Whitney Test and Friedman Test (Nonparametric Repeated-Measures ANOVA).

With regard to the PP-protocol of TMS, a significant difference was found between migraineurs and HCs only in the SICI at a 3 ms ISI only at baseline (t0) (U = 92; *p* = 0.04). Nevertheless, no significant differences were found between migraineurs and HCs at t1 (U = 86; *p* = 0.1) and at t2 (U = 67; *p* = 0.6). In particular, at t0, the amplitude of MEP at a 3 ms ISI was 0.3 (SD ± 0.5) for migraineurs and 0.05 (SD ± 0.03) for HCs. The SICI amplitude in migraineurs at a 3 ms ISI decreased at t1 (0.2 ± 0.2) and at t2 (0.1 ± 0.2). In addition, the Friedman Test showed statistically significant differences (^χ2^_F_(2) = 6.465; *p* = 0.03) among the three evaluations in the amplitude of SICI at a 3 ms ISI (t0 vs. t1 3.500, ns *p* > 0.05; t0 vs. t2 11.500, * *p* < 0.05; t1 vs. t2 8.000, ns *p* > 0.05). Moreover, no differences were found in the SICI amplitude at a 5 ISI and in the amplitude of intracortical facilitation (ICF) at 10, 15, and 20 ms ISIs between migraineurs and HCs, nor among the three evaluations (Figure 2a,b) (Table 2).

### 3.2. Pressure Pain Threshold

Migraine patients had a lower PPT compared to HCs in all muscles assessed except for the temporalis left and procerus. The PPT increased in all muscles at t1 and t2 and non-statistical differences were found between migraineurs and HCs at t0 and t2, respectively, in the Mann–Whitney Test. The Friedman Test revealed statistically significant differences among the three assessments in the sub-occipitalis left (^χ2^_F_(2) = 11.455; *p* = 0.002; Kendall’s W = 0.9990; t0 vs. t1—12.000, * *p* < 0.05; t0 vs. t2—15.000, ** *p* < 0.01; t1 vs. t2—3.000, ns *p* > 0.05) and in the trapezius right (^χ2^_F_(2) = 13.636; *p* = 0.0004; Kendall’s W = 1.0000; t0 vs. t1—15.000, ** *p* < 0.01; t0 vs. t2—15.000, ** *p* < 0.01; t1 vs. t2 0.000, ns *p* > 0.05). On the other hand, no differences were found among the three evaluations in the temporalis left (^χ2^_F_(2) = 0.7273; *p* = 0.7; Kendall’s W = 0.5620) and right (^χ2^_F_(2) = 0.7273; *p* = 0.7; Kendall’s W = 0.9990), in the sub-occipitalis right (^χ2^_F_(2) = 5.091; *p* = 0.08; Kendall’s W = 1.0000), in the masseter left (^χ2^_F_(2) = 0.1818; *p* = 0.9; Kendall’s W = 1.0000) and right (^χ2^_F_(2) = 4.545; *p* = 0.11; Kendall’s W = 1.0000), in the trapezius left (^χ2^_F_(2) = 5.091; *p* = 0.08; Kendall’s W = 1.0000), in the procerus (^χ2^_F_(2) = 5.163; *p* = 0.07; Kendall’s W = 0.9990), and in the tensor fasciae latae left (^χ2^_F_(2) = 1.273; *p* = 0.6; Kendall’s W = 1.0000) and right (^χ2^_F_(2) = 1.273; *p* = 0.6; Kendall’s W = 1.0000) (Table 3).

### 3.3. Headache Parameters

All headache parameters improved at t1 and t2 (Table 1). In particular, the mean MMDs decreased from 15 (SD ± 3) days at t0 to 7 (SD ± 4) at t1 and to 6 (SD ± 3) at t2. The Friedman Test found statistically significant differences among the three evaluations (^χ2^_F_(2) = 17.636; *p* ≤ 0.0001; Kendall’s W = 0.4671; t0 vs. t1 14.000, * *p* < 0.01; t0 vs. t2 19.000 *** *p* < 0.001; t1 vs. t2 5.000, ns *p* > 0.05). At t1, 6 patients were responders (of which 2 were super-responders), and at t2, 8 patients were responders (of which 2 were super-responders). The average duration of attacks significantly decreased from 164 h (SD ± 181) at t0 to 44 h (SD ± 35) at t1 and to 36 h (SD ± 31) at t2. Furthermore, the variance among the three assessments was statistically significant (^χ2^_F_(2) = 14.364; *p* = 0.0002; Kendall’s W = 0.6712; t0 vs. t1 13.000, ns *p* > 0.05; t0 vs. t2 17.000, ** *p* < 0.01; t1 vs. t2 4.000, ns *p* > 0.05).

Mean Monthly Drug Intake decreased from 15 (SD ± 7) at t0 to 7 (SD ± 3) at t1 and to 6 (SD ± 3) at t2. Moreover, the variance shown by the Friedman Test was statistically significant (^χ2^_F_(2) = 11.762; *p* = 0.002; Kendall’s W = 0.6290; t0 vs. t1 10.000, ns *p* > 0.05; t0 vs. t2 15.500, ** *p* < 0.01; t1 vs. t2 5.500, ns *p* > 0.05). Lastly, the disability related to headache assessed with the MIDAS questionnaire significantly reduced from 100 (SD ± 25) to 21 (SD ± 15) points (*p* = 0.001, Wilcoxon matched-pairs).

## 4. Discussion

The main findings of this study are the significant effects of anti-CGRP mAb treatment on the TMS parameters of intracortical inhibition and the rise in the resting motor threshold in our group of patients affected by resistant migraine. The clinical effect of therapy on migraine is associated with the increase in short-interval intracortical inhibition (SICI), resting motor threshold (RMT), and Pressure Pain Threshold (PPT). In all patients, all clinical headache parameters improved significantly 3 months after the first injection of mAbs and the improvement was maintained at the 1-month follow-up. At baseline, migraineurs and HCs had significant differences in all TMS parameters and in PPT, while at follow-up assessment, no differences were observed on RMT, SICI, and PPT between the two groups.

RMT reflects different aspects of brain circuit excitability investigated by SS-TMS [14,34,35]. We found that RMT significantly increased in the migraine group after anti-CGRP mAb treatment, reaching the same values of HCs. These observations may suggest that anti-CGRP mAbs may act not only peripherally but also at a central level, probably on cortico-cortical axons excitability or on fast synaptic transmission. Recent studies suggest that anti-CGRP mAbs do not penetrate the blood–brain barrier. They may exert an indirect effect on the trigeminocervical complex, on the meningeal vessels, or on the hypothalamus [20,21]. The trigeminocervical complex represents the main actor of an interplay between central and peripheral structures [2,3,4]. Thanks to these connections, anti-CGRP mAbs may also have a central effect. In fact, peripheral sensitization of the trigeminocervical complex leads to increased painful stimulation of the thalamus and consequently to central sensitization [20]. Furthermore, the hypothalamus plays an important role both in migraine pathogenesis and in descending pain inhibition [36,37]. The CGRP receptor is expressed also at the hypothalamic level, where the capillary endothelium is fenestrated. Consequently, authors have supposed a central effect of anti-CGRP mAbs on migraine [38,39]. As already reported with some symptomatic medication, in particular triptans [30,40], the improvement in migraine after anti-CGRP mAb treatment could be related to a normalization of cortical excitability and pain perception. The increase in motor threshold in sensory motor areas may be an indirect epiphenomenon of action of anti-CGRP mAbs on the trigeminocervical complex and on the hypothalamus.

SICI and ICF allow the assessment of the GABAergic inhibitory circuits and the glutamatergic excitatory pathways of neurotransmission, respectively [34,35,41]. Neurophysiological studies in migraine evidenced a higher reduction in SICI, reflecting the lack of habituation during stimulus repetition, and a more pronounced ICF, reflecting sensitization during stimulus repetition compared with HCs [14,15,42]. No previous studies investigated SICI and ICF before and after anti-CGRP mAb treatment. Our results highlighted significant differences between migraineurs and HCs in SICI only at a 3 ms ISI, but not at a 5 ms ISI, while no differences were found at 10 ms, 15 ms, or 20 ms of ICF. One unexpected finding was that at the end of the 4 months of anti-CGRP mAbs, no differences were found between migraineurs and HCs in SICI at a 3 ms ISI, which may be related to a normalizing effect on inhibitory circuits mediated by GABAergic neurotransmission. The increase in intracortical inhibition, associated with the rise in motor threshold, suggests a combined and probably indirect effect of anti-CGRP mABs on GABAergic neurotransmission and cortico-cortical axon excitability, which are usually abnormal in migraine. Consequently, it could be supposed that anti-CGRP mAbs may act directly or indirectly on the central nervous system even in the case of a lack of habituation and of sensitization. Moreover, the lack of habituation depends on thalamocortical dysrhythmia. The response of the thalamus to inputs of the trigeminocervical complex is mediated by CGRP antagonists, which block the release of CGRP in areas involved in migraine through the inhibition of neurogenic inflammation [22,23,24,25,26]. Understanding whether long-term treatment with monoclonal antibodies would result in a normalization of SICI also at a 5 ms ISI would be compelling, as well as whether this normalization would last over time.

We found a significantly reduced PPT in almost all points of the craniocervical regions and also in the extra-cephalic regions. Several pieces of research highlighted a lower PPT in migraineurs compared to HCs both in the trigeminocervical complex and throughout the body [18,33,43]. These parameters have never been studied in patients with migraine after mAb treatment. One study assessed PPT in CM after Onabotulinumtoxin-A (BoNT-A), physical therapy, and their combination [11]. Although each treatment increased PPT, the combined approach was more effective than the respective monotherapies. Different pharmacological and non-pharmacological therapies target sub-occipitalis muscles in migraine due to their anatomical connections [17,18,33]. Similarly to BoNT-A in monotherapy, anti-CGRP mAbs in monotherapy increased PPT bilaterally in the sub-occipitalis muscles, too [18]. The sub-occipitalis muscles are innervated both by the C1 nerve and by the greater occipital nerve. Particularly, the rectus capitis posterior is anatomically linked to the dura mater, which in turn is innervated by the ophthalmic division of the trigeminal nerve, too [44]. The increase in PPT over craniocervical and extracervical regions suggests a pain modulation effect of anti-CGRP mAbs, which is also related to the improvement in headache parameters [21]. In fact, our patients reported a progressive and significant reduction in MMDs, duration of attacks, symptomatic drug intake, and MIDAS. At t2, 5 out of 8 responder patients were super-responders. This high rate of responders reflects what has already been found in meta-analyses by evaluating the efficacy and tolerability of anti-CGRP mAbs in the preventive treatment of EM and CM [45,46].

Our neurophysiological outcomes on RMT, SICI, and PPT corroborate the hypothesis that anti-CGRP mAbs may directly or indirectly normalize cortical excitability and PPT through a reduction in peripheral and central sensitization. Despite these promising results, further studies are needed to establish whether anti-CGRP mAbs may also exert an effect on the lack of habituation. The exact site and mechanism of action of this targeted therapy are still debated. Recent studies reported a possible central effect of erenumab and galcanezumab; in fact, a decrease in hypothalamic activation was found among patients treated with these two mAbs [47]. On such a basis, our findings suggest some hypotheses on functioning: (1) an indirect peripheral modulation through trigeminal afferents fibers leads to a normalization of cortical excitability and PPT; (2) a direct central modulation through the thalamus and hypothalamus influences pain perception and central sensitization; and (3) an indirect central effect through the reduction in analgesic intake, in particular triptans, influences cortical excitability and pain perception [30,37,40].

Our study had some limitations. First, the small sample size did not allow a stratification between HFEM and CM, nor between responders and partial responders, nor among the different anti-CGRP mAbs used. However, our aim was to evaluate the neurophysiological effect of monoclonal antibodies on central and peripheral outcomes, since the effectiveness on clinical parameters had already been demonstrated. Second, a long-term follow-up is necessary to determine whether early results last over time. Third, the guidelines allow the consumption of symptomatic medications in the case of severe migraine attack, but this intake may bias the results. Despite these limitations, the study presented three strong points: (1) for the first time, the cortical excitability was studied with TMS after anti-CGRP mAb treatment; (2) for the first time, the changes in PPT were assessed with a specific algometer after anti-CGRP mAb treatment; and (3) for the first time, the neurophysiological outcomes on cortical excitability and PPT between migraineurs and HCs before and after anti-CGRP mAb treatment were compared.

## 5. Conclusions

This study evidences an abnormal cortical excitability in migraineurs, reflected by a lower RMT and SICI, as well as hyperalgesia, reflected by a lower PPT in the cephalic and extra-cephalic muscles, compared to HCs. Despite the site and mechanism of action still being uncertain, anti-CGRP mABs seem to be able to modulate the central and peripheral sensitization to pain both indirectly through the trigeminocervical complex and directly through the thalamus or hypothalamus. Lastly, this study highlights the clinical and neurophysiological effects of anti-CGRP monoclonal antibodies in migraine. In the future, larger randomized controlled trials may shed light on the possible responses to each anti-CGRP mAb and their effect on the habituation phenomenon.

## Figures and Tables

**Table 2:** Paired-Pulse-TMS in migraine group at t0, t1, and t2 compared to healthy controls.

PP-TMS	M Mean (SD) Median (IQR)	HC Mean (SD) Median (IQR)	Differences t0 vs. t1 vs. t2	Differences M vs. HC
3 ms (SICI)				
	t0 0.3 (SD ± 0.5) 0.1 (0.1–0.25)	0.05 (SD ± 0.03) 0.05 (0.02–0.09)	t0 vs. t1: ns	U = 92; *p* = 0.04 *
	t1 0.2 (SD ± 0.2) 0.1 (0.35–0.25)		t0 vs. t2: *p* < 0.05 *	U = 86; *p* = 0.1
	t2 0.1 (SD ± 0.2) 0.04 (0.01–0.07)		t1 vs. t2: ns	U = 67; *p* = 0.6
5 ms (SICI)				
	t0 0.6 (SD ± 1.4) 0.1 (0.06–0.3)	0.1 (SD ± 0.1) 0.2 (0.01–0.3)	t0 vs. t1: ns	U = 62; *p* = 0.9
	t1 0.4 (SD ± 0.5) 0.2 (0.1–0.5)		t0 vs. t2: ns	U = 72; *p* = 0.4
	t2 0.4 (SD ± 0.5) 0.3 (0.06–0.85)		t1 vs. t2: ns	U = 79; *p* = 0.2
10 ms (ICF)				
	t0 0.9 (SD ± 1) 0.8 (0.1–1.3)	1.1 (SD ± 1) 1 (0.25–1.4)	t0 vs. t1: ns	U = 73.5; *p* = 0.4
	t1 1.2 (SD ± 1.1) 1 (0.5–1.9)		t0 vs. t2: ns	U = 67.5; *p* = 0.6
	t2 0.8 (SD ± 0.9) 0.5 (0.15–0.9)		t1 vs. t2: ns	U = 75.5; *p* = 0.3
15 ms (ICF)				
	t0 0.9 (SD ± 1.2) 0.5 (0.14–1.15)	0.9 (SD ± 0.9) 0.9 (0.2–1.4)	t0 vs. t1: ns	U = 72.5; *p* = 0.4
	t1 1.6 (SD ± 1.2) 1.7 (0.75–2.2)		t0 vs. t2: ns	U = 82.5; *p* = 0.1
	t2 1 (SD ± 0.9) 0.5 (0.35–1.3)		t1 vs. t2: ns	U = 62.5; *p* = 0.9
20 ms (ICF)				
	t0 1 (SD ± 1.2) 0.5 (0.19–1.3)	0.8 (SD ± 0.6) 0.5 (0.25–1.4)	t0 vs. t1: ns	U = 61; *p* = 0.9
	t11.4 (SD ± 1.2) 0.9 (0.55–1.9)		t0 vs. t2: ns	U = 76.5; *p* = 0.3
	t2 0.8 (SD ± 1) 0.3 (0.2–1.25)		t1 vs. t2: ns	U = 63.5; *p* = 0.8
RMT				
	t0 59.2 (SD ± 12.3) 62 (53.5–68)	68.6 (SD ± 8.1) 70 (65–75)	t0 vs. t1: *p* < 0.05 *	U = 91; *p* = 0.04 *
	t1 66 (SD ± 12.3) 67 (60.5–74)		t0 vs. t2: ns	U = 69.5; *p* = 0.5
	t2 70 (SD ± 15.8) 70 (57–84.5)		t1 vs. t2: ns	U = 64.5; *p* = 0.8

* *p* < 0.05. Friedman Test (Nonparametric Repeated-Measures ANOVA) (t0), after 3 months of therapy with monoclonal antibodies (t1), and after 1 month of follow-up (t2); Mann–Whitney test between migraineurs (M) and healthy controls (HCs). PP-TMS: Paired-Pulse Transcranial Magnetic Stimulation; SICI: short-interval intracortical inhibition; ICF: intracortical facilitation; RMT: resting motor threshold.

**Table 3:** Pressure Pain Threshold in migraine group at t0, t1, and t2 compared to healthy controls.

PPT	M Mean (SD) Median (IQR)	HC Mean (SD) Median (IQR)	Differences t0 vs. t1 vs. t2	Differences M vs. HC
Temporalis left	t0 250.6 (SD ± 118) 346.9 (258–443.5)	285.5 (SD ± 116) 260 (211–347.8)	t0 vs. t1: ns	U = 70; *p* = 0.5
	t1 285 (SD ± 138.8) 246.3 (209–325.6)		t0 vs. t2: ns	U = 65; *p* = 0.7
	t2 273.2 (SD ± 94.5) 237.1 (204.5–325.6)		t1 vs. t2: ns	U = 64; *p* = 0.8
Temporalis right	t0 251.7 (SD ± 108.2) 319.4 (233.9–404)	315.6 (SD ± 65.3) 328 (264.1–354.1)	t0 vs. t1: ns	U = 91; *p* = 0.04 *
	t1 270 (SD ± 81.7) 268.5 (222.7–292)		t0 vs. t2: ns	U = 84; *p* = 0.1
	t2 255 (SD ± 75.8) 242.3 (202.2–291.7)		t1 vs. t2: ns	U = 89; *p* = 0.06
Sub-occipitalis left	t0 257.2 (SD ± 109.8) 246.9 (195.3–334.4)	563.7 (SD ± 668.5) 314.6 (295.4–475.6)	t0 vs. t1: *p* < 0.05 *	U = 91; *p* = 0.04 *
	t1 345.3 (SD ± 77.1) 339.7 (279.3–377.2)		t0 vs. t2: *p* < 0.01 **	U = 73; *p* = 0.4
	t2 341.2 (SD ± 96.7) 340.3 (285.2–379.2)		t1 vs. t2: ns	U = 69; *p* = 0.6
Sub-occipitalis right	t0 241.4 (SD ± 109.5) 235.8 (172.4–310.8)	318.1 (SD ± 64.6) 299.2 (283.7–344.9)	t0 vs. t1: ns	U = 91; *p* = 0.04 *
	t1 309.2 (SD ± 84.2) 312.3 (249.9–379.5)		t0 vs. t2: ns	U = 65; *p* = 0.7
	t2 318.6 (SD ± 86.7) 326 (273.7–347.8)		t1 vs. t2: ns	U = 66; *p* = 0.7
Masseter left	t0 214.7 (SD ± 93.4) 207.1 (159.7–234.5)	257.9 (SD ± 41.5) 255.5 (232.6–266.6)	t0 vs. t1: ns	U = 91; *p* = 0.04 *
	t1 219.9 (SD ± 63.4) 209 (192.7–243.7)		t0 vs. t2: ns	U = 91; *p* = 0.04 *
	t2 232.9 (SD ± 86.7) 230.6 (182.2–249.9)		t1 vs. t2: ns	U = 89; *p* = 0.06
Masseter right	t0 171.2 (SD ± 59.3) 170.3 (119.8–220.1)	255.4 (SD ± 68.7) 235.2 (211–305.4)	t0 vs. t1: ns	U = 99; *p* = 0.01 *
	t1 184.6 (SD ± 46.6) 180.3 (148.6–194.6)		t0 vs. t2: ns	U = 103; *p* = 0.004 *
	t2 218.2 (SD ± 79.6) 209.1 (166.5–225.1)		t1 vs. t2: ns	U = 85; *p* = 0.1
Trapezius left	t0 334.8 (SD ± 146.4) 346.9 (258–443.5)	509.1 (SD ± 180.2) 471 (369.1–597)	t0 vs. t1: ns	U = 93; *p* = 0.03 *
	t1 398.1 (SD ± 114.1) 369.5 (322.4–463.8)		t0 vs. t2: *p* < 0.05 *	U = 83; *p* = 0.1
	t2 423.11 (SD ± 145.7) 369.9 (324.9–509.4)		t1 vs. t2: ns	U = 78; *p* = 0.2
Trapezius right	t0 321.7 (SD ± 125.2) 319.4 (233.9–404)	522.8 (SD ± 171.9) 499.8 (409.9–600)	t0 vs. t1: *p* < 0.01 **	U = 100; *p* = 0.008 *
	t1 462.5 (SD ± 104.9) 479.5 (395.6–543.1)		t0 vs. t2: *p* < 0.01 **	U = 70; *p* = 0.5
	t2 473.6 (SD ± 203.5) 454.7 (318.1–616.7)		t1 vs. t2: ns	U = 70; *p* = 0.5
Procerus	t0 250.7 (SD ± 82.3) 246.9 (204.1–317.5)	287.4 (SD ± 83.6) 285.5 (230.3–343.5)	t0 vs. t1: ns	U = 74; *p* = 0.4
	t1 308.7 (SD ± 75.4) 294 (275–338.1)		t0 vs. t2: ns	U = 68; *p* = 0.6
	t2 328 (SD ± 55.9) 310.3 (280.3–382.5)		t1 vs. t2: ns	U = 67; *p* = 0.6
Tensor fasciae latae left	t0 490.8 (SD ± 238.5) 484.8 (339.4–563.1)	667.7 (SD ± 182.3) 642.9 (574.9–768.7)	t0 vs. t1: ns	U = 92; *p* = 0.04 *
	t1 560.2 (SD ± 217.6) 494.5 (409–731.1)		t0 vs. t2: ns	U = 79; *p* = 0.2
	t2 590.1 (SD ± 255.7) 510.2 (418.7–719.6)		t1 vs. t2: ns	U = 79; *p* = 0.2
Tensor fasciae latae right	t0 486.6 (SD ± 235.2) 463.2 (259.3–651.4)	1283.9 (SD ± 1891.8) 710.6 (584.5–917.6)	t0 vs. t1: ns	U = 95; *p* = 0.02 *
	t1 558.2 (SD ± 235.5) 495.8 (401.4–730.4)		t0 vs. t2: ns	U = 89; *p* = 0.06
	t2 567.3 (SD ± 260.5) 658.5 (354.7–682)		t1 vs. t2: ns	U = 85; *p* = 0.1

* *p* < 0.05; ** *p* < 0.01. Friedman Test (Nonparametric Repeated-Measures ANOVA) (t0), after 3 months of therapy with monoclonal antibodies (t1), and after 1 month of follow-up (t2); Mann–Whitney Test between migraineurs (M) and healthy controls (HCs). PPT: Pressure Pain Threshold.

## Data Availability

The principal author takes full responsibility for the data presented in this study, analysis of the data, conclusions, and conduct of the research. The dataset page containing authors’ details analyzed during the current study are available from the corresponding author on reasonable request.

## Abbreviation

anti-CGRP mAbs	Anti-Calcitonin Gene-Related Peptide monoclonal Antibodies
BoNTA	Onabotulinumtoxin-A
CGRP	Calcitonin Gene-Related Peptide
CSP	Cortical Silent Period
CM	Chronic Migraine
EM	Episodic Migraine
FDA	Food and Drug Administration
HF-EM	High-Frequency Episodic Migraine
ICHD-3	International Classification of Headache Disorders, Third Edition
ISI	interstimulus interval
ICF	intracortical facilitation
LICI	long-interval intracortical inhibition
LF-EM	Low-Frequency Episodic Migraine
MOH	Medication Overuse Headache
MEP	motor-evoked potential
NSAIDs	Nonsteroidal Anti-Inflammatory Drugs
PPT	Pressure Pain Threshold
PP-TMS	Paired-Pulse Transcranial Magnetic Stimulation
RCT	Randomized Control Trial
RMT	resting motor threshold
SICI	short-interval intracortical inhibition
SP-TMS	Single-Pulse Transcranial Magnetic Stimulation
TMS	Transcranial Magnetic Stimulation
